# Tissue-resident *Klebsiella quasipneumoniae* contributes to progression of idiopathic pulmonary fibrosis by triggering macrophages mitophagy in mice

**DOI:** 10.1038/s41420-025-02444-6

**Published:** 2025-04-12

**Authors:** Chunjie Xu, Peiyi Sun, Qiyue Jiang, Yao Meng, Luyao Dong, Xiukun Wang, Xinxin Hu, Congran Li, Guoqing Li, Ruifang Zheng, Xuefu You, Xinyi Yang

**Affiliations:** 1 Beijing Key Laboratory of Technology and Application for Anti-Infective New Drugs Research and Development/ Laboratory of Pharmacology, Institute of Medicinal Biotechnology, Chinese Academy of Medical Sciences & Peking Union Medical College, 100050 Beijing, China; 2 Division for Medicinal Microorganism-Related Strains, CAMS Collection Center of Pathogenic Microorganisms, 100050 Beijing, China; 3 State Key Laboratory of Bioactive Substances and Functions of Natural Medicines, Institute of Medicinal Biotechnology, Chinese Academy of Medical Sciences & Peking Union Medical College, 100050 Beijing, China; 4 Department of Occupational Health and Environmental Health, School of Public Health, Capital Medical University, 100069 Beijing, China; 5 State Key Laboratory of Respiratory Health and Multimorbidity, Institute of Medicinal Biotechnology, Chinese Academy of Medical Sciences & Peking Union Medical College, 100050 Beijing, China; 6 Xinjiang Key Laboratory of Uygur Medical Research, Xinjiang Institute of Materia Medica, Urumqi, 841100 China

**Keywords:** Cell delivery, Mitophagy

## Abstract

Idiopathic pulmonary fibrosis (IPF) is a progressive and chronic interstitial lung disease with unclear underlying pathogenic mechanisms. Dysbiosis of the lung microbiota is believed to be associated with the development of fibrosis; however, the roles of the microbiome in the respiratory functions of hosts with IPF remain poorly understood. To investigate the relationship between the lung microbiome and the pathological processes of idiopathic pulmonary fibrosis under laboratory conditions, C57BL/6 J mice were exposed to bleomycin and observed at 7, 14, 21, and 28 days post-exposure. 16S rDNA analysis revealed that the lung microbial community exhibited dysbiosis in the bleomycin-induced pulmonary fibrosis model, characterized by an abnormally high proportion of *Klebsiella quasipneumoniae* (*K. quasipneumoniae*), as confirmed by RNA fluorescence in situ hybridization. Throughout the progression of experimental pulmonary fibrosis, Tax4Fun analysis indicated that the abundance of *K. quasipneumoniae* differed significantly between model mice and control mice, correlating with the sustained activation of reactive oxygen species (ROS) pathways. Importantly, the dysbiosis of *K. quasipneumoniae* may serve as a critical factor triggering increased ROS levels, accompanied by macrophage mitophagy, ultimately leading to the overexpression of TGF-β1, a key player in the pathogenesis of pulmonary fibrosis. These findings suggest that lung microbiota dysbiosis exacerbates the progression of bleomycin-induced pulmonary fibrosis related to macrophage mitophagy.

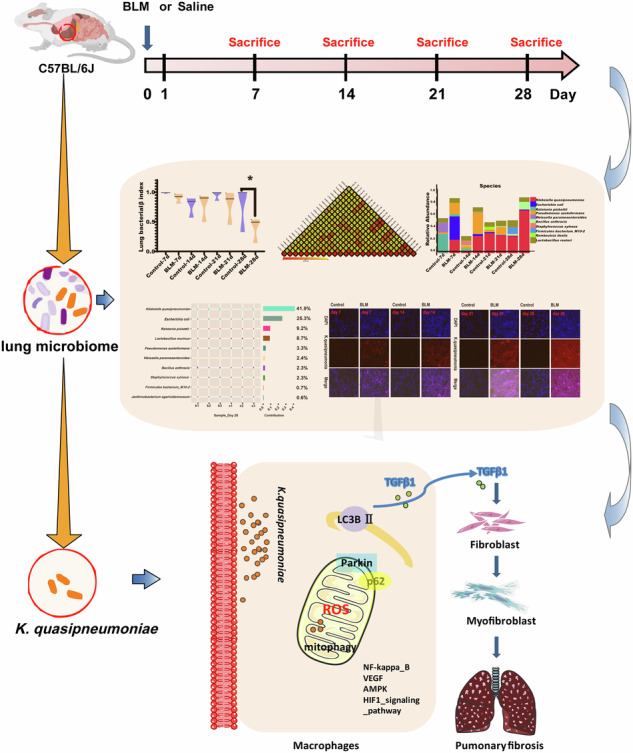

## Introduction

Idiopathic pulmonary fibrosis (IPF) is an interstitial lung disease of unknown etiology, characterized by chronic and progressive scarring in the lung parenchyma. This aberrant scarring leads to restrictive ventilatory impairment, reduced lung function, and eventually respiratory failure, which is typically fatal [1, 2]. Although the mechanisms underlying fibrosis in IPF remain unclear, it is believed that recurrent micro-injuries from external triggers such as cigarette smoke, gastroesophageal reflux, dust particles, viral infections, or alterations in the lung microbiome play a significant role. These injuries result in dysfunction of the alveolar epithelium, triggering an abnormal wound-healing response that is characterized by excessive deposition of extracellular matrix (ECM), ultimately leading to fibrosis [3].

Pulmonary fibrosis (PF) means irreversible changes to architecture and function of the lung, involving local alterations in alveolar mucosa and airway, blood flow, oxygen tension, and dysregulation in host immunity. The anatomical, physiological, and immunological characteristics of patients with PF may exert selective pressure on the bacterial communities in their lower respiratory tract, leading to microbial dysbiosis or changes in the microbiome [4]. Although the role of bacteria in IPF pathogenesis is still not fully understood, there is growing evidence linking IPF to alterations in the lung microbiome. With the recent use of culture-independent techniques for microbiological analysis, such as RNA in situ hybridization (ISH), and high-throughput 16S ribosomal DNA (rDNA) sequencing, previously unrecognized changes in the lung microbiome and increased bacterial burden in the bronchoalveolar lavage (BAL) fluid of IPF patients have been identified [5]. Several studies involving medium to large cohorts of IPF patients have shown that increased bacterial burden in the lungs is associated with disease progression and may serve as an independent predictor of mortality [5, 6]. While the correlation between bacterial burden and IPF pathogenesis does not imply causation, it is widely believed that bacteria in the distal airways have the potential to cause persistent or repetitive injury to the alveolar epithelial cells, either directly or indirectly, through the host immune response. The lung microbiota, through its interaction with the immune system, may initiate a cascade of events leading to fibrosis. Microbial infections, by altering the microbial community, could disrupt the initiation or perpetuation of fibrosis, as evidenced by findings that immunosuppressive therapy decreases the progression-free survival of IPF patients [6]. However, the exact role of lung microbiota alterations in IPF progression remains to be elucidated.

Alveolar macrophages (AMs), the first line of defense against respiratory pathogens [7], play an integral role in the pathogenesis of PF by initiating immune responses and generating reactive oxygen species (ROS). These immune activities are essential for maintaining homeostasis, immune surveillance, cellular debris removal, microbial clearance, and inflammation resolution [8]. Mitochondria, the energy-producing powerhouses within cells, are particularly vulnerable to the detrimental effects of oxidative stress caused by ROS. While the processes involved in lung remodeling during PF are poorly understood, ROS produced by alveolar macrophages are thought to be crucial for fibrosis development, primarily through the increased expression of transforming growth factor-β1 (TGF-β1) [9]. Studies have shown that reducing mitochondrial oxidative stress can decrease TGF-β1 levels and slow the progression of PF in mice [10]. Although myofibroblasts are well-known to drive fibrosis, macrophages are also critical players, promoting fibroblast activity, increasing their proliferation, and stimulating excessive ECM production, all of which exacerbate IPF progression [11]. However, the interaction between AMs and the lung microbiota in IPF patients is yet to be known, and further investigation is needed to determine whether lung microbiota affects IPF progression through AMs.

To address these issues, this study utilized 16S rDNA amplicon sequencing to examine changes in the lung microbiota of a bleomycin (BLM)-induced pulmonary fibrosis model. RNA ISH was used to identify which bacterial species play a significant role in the development of BLM-induced PF. Based on bacterial genera identified in microbiome studies, further experiments were conducted to establish causality and mechanistic links to disease progression, with the goal of investigating how the lung microbial community influences fibrosis progression in mice with BLM-induced PF.

## Results

**Fig. 1:**
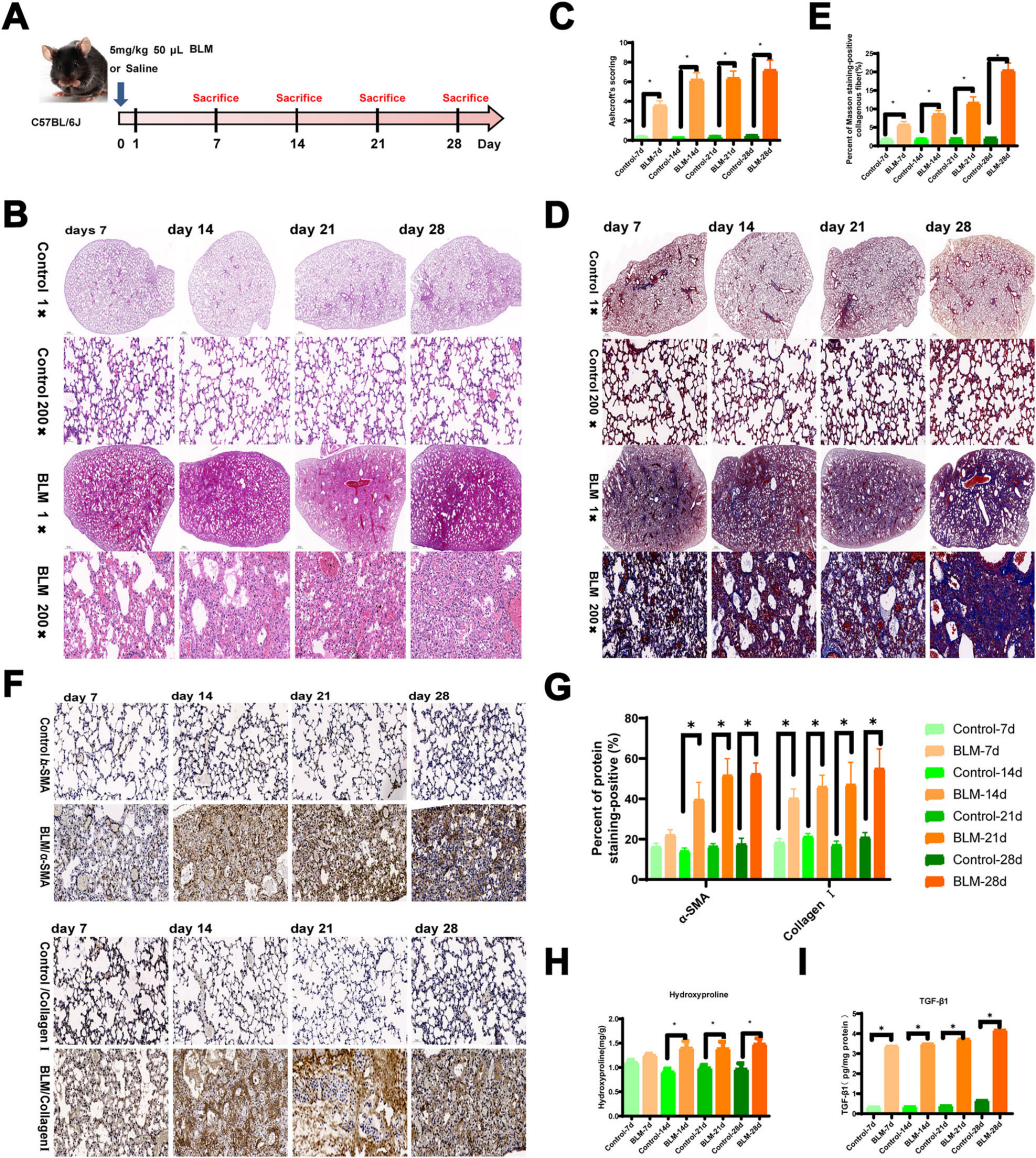
The representative results of lung tissue in mice with PF at four time points after BLM induction. **A** The experimental process of mice instilled with BLM. **B**, **C** HE staining and Ashcroft scores. **D**, **E** Masson staining of mouse lung tissue at four time points. **F**, **G** IHC was performed, and the levels of α-SMA and collagen I were determined in the lungs of mice. **H** Hydroxyproline detection in the mouse lung tissue at four time points. **I** Active TGF-β1 was measured in BAL fluid of mice from Control and BLM groups at four time points by ELISA. Scale bar, 500 μm and 50 μm. (*n* = 3 per group). *P < 0.05 compared with the control group.

To evaluate the pathological changes in mice, C57BL/6 mice were administered 0.05 mL of BLM at a dose of 5 mg/kg and sacrificed at different time points (7, 14, 21, and 28 days) post-exposure (Fig. 1A). Hematoxylin-eosin (HE) staining revealed normal alveolar structure and septa with no evidence of edema, inflammation, fibrosis, or exudation in the control group (Fig. 1B). However, by day 7 following BLM exposure, inflammatory cell infiltration was occasionally observed in the lung tissue, with some alveolar septal widening but no significant changes in the bronchial walls. As exposure duration increased, more inflammatory cells accumulated in the alveoli, accompanied by bronchial wall thickening, significant alveolar septal widening, extensive alveolar collapse, and increased compensatory alveolar dilation on days 14, 21, and 28 (Fig. 1B). Additionally, the fibrotic area and fibrosis score increased significantly with extended BLM exposure (Fig. 1C).

Hydroxyproline levels, indicative of collagen deposition, were significantly elevated in the lung tissues of BLM-treated mice over time (Fig. 1H). Compared to controls, increased blue collagen deposition and more pronounced pulmonary fibrosis (PF) symptoms were observed in BLM-exposed mice (Fig. 1D, E). Multiple tissue microarrays confirmed that the extracellular matrix (ECM) consisted of α-SMA and major fibrillar collagens [12]. Immunohistochemical analysis (IHC) showed significant upregulation of α-SMA and collagen I on days 14, 21, and 28, although there was no significant change on day 7 (Fig. 1F, G). Active TGF-β1 levels measured in the bronchoalveolar lavage (BAL) fluid were higher in BLM-treated mice compared to controls at all time points (Fig. 1I). Forced oscillation technique was used to assess lung function, revealing a significant decline after BLM exposure (Supplementary Fig. S1). These results indicate that BLM induces PF in mice, with fibrosis worsening over time.

**Fig. 2:**
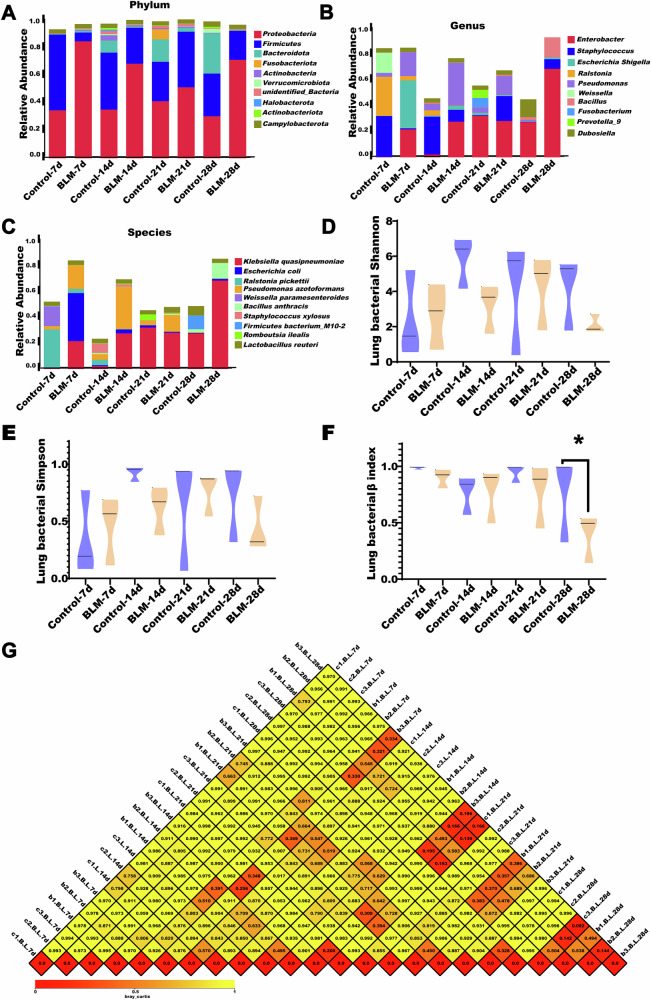
Changes of lung microbiota in mice of BLM-induced PF at different times. **A**–**C** Relative abundance of lung microbiota based on the level of phylum, genus and species (top10). **D**, **E** Assessing alpha diversity of lung microbiota among groups. **F**, **G** The beta-diversity of lung microbiota among groups. *P < 0.05 compared with the control group, *n* = 3 per group.

The relative abundance of the top 10 bacterial taxa in BAL fluid on days 7, 14, 21, and 28 is shown in Fig. 2A–C. At the phylum level, *Proteobacteria* and *Firmicutes* dominated at all time points, with *Proteobacteria* significantly more abundant in the BLM group compared to controls (Fig. 2A). At the genus level, *Enterobacter* and *Staphylococcus* were the dominant taxa, fluctuating over time, with *Enterobacter* more abundant in the BLM group (Fig. 2B). At the species level, *K. quasipneumoniae* and *Escherichia coli* were prevalent, with *K. quasipneumoniae* increasing significantly in the BLM group over time (Fig. 2C).

Alpha diversity, assessed using the Shannon and Simpson indices, showed no significant difference between groups, indicating similar species diversity and evenness (Fig. 2D, E). Beta diversity, however, revealed differences between the microbial communities of BLM-treated and control mice, with significant changes observed on day 28 (*P* < 0.05) (Fig. 2F, G). These findings suggest that the lung microbiota was in a dynamic state but exhibited an imbalance in the dominant taxa following BLM treatment.

**Fig. 3:**
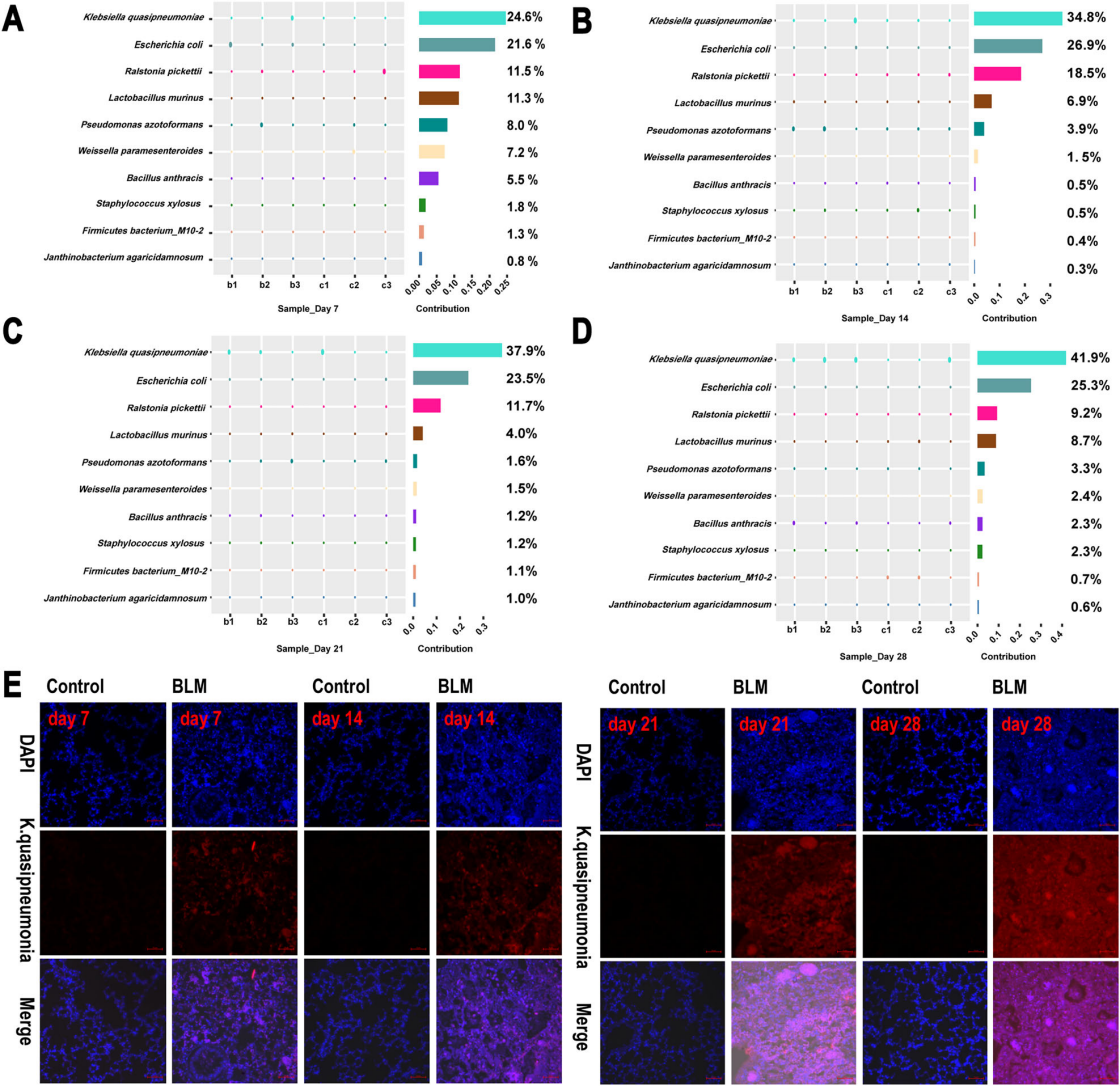
*K. quasipneumoniae* 16S rRNA expression in lung tissue of the mice at four time points. **A**–**D** Simper analysis at species level revealed that the largest contribution of intergroup differences (top1 ~ 10: *Klebsiella quasipneumoniae*, *Escherichia coli*, *Ralstonia pickettii*, *Lactobacillus murinus*, *Pseudomonas azotoformans*, *Weissella paramesenteroides*, *Bacillus anthracis*, *Staphylococcus xylosus*, *Firmicutes bacterium_M10-2*, *Janthinobacterium agaricidamnosum*). **E** *K. quasipneumoniae* 16S rRNA (red) expression in the FFPE mouse lung with RNA scope™ Fluorescent Direction Assay v2. Nuclei were labeled with DAPI (blue). *n* = 3 per group.

To quantify the contribution of each species to the differences between groups, the Simper index was used to decompose the Bray-Curtis dissimilarity index. Results showed that *K. quasipneumoniae* accounted for the largest difference between BLM and control groups on days 7 and 14, contributing 24.6% and 34.8%, respectively (Fig. 3A–D). This difference further increased to 37.9% and 41.9% on days 21 and 28, respectively. 16S rRNA fluorescence in situ hybridization (FISH) was used to observe dynamic changes in *K. quasipneumoniae* in lung tissue, with increasing fluorescence intensity over time after BLM exposure (Fig. 3E). Thus, the proportion of *K. quasipneumoniae* increased as BLM exposure progressed.

**Fig. 4:**
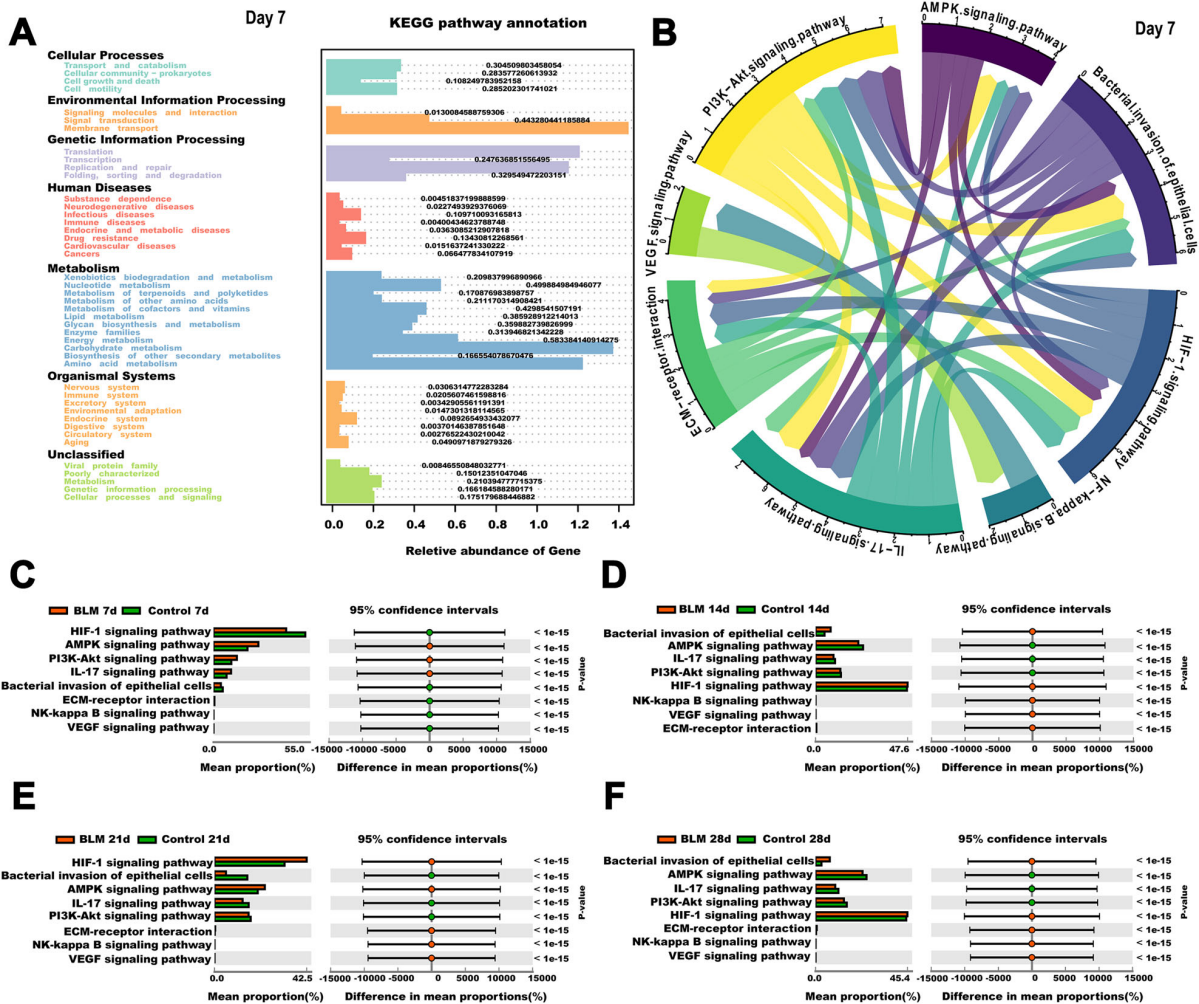
Functional analysis of lung microbiota in mice of BLM-induced lung fibrosis. **A** The prediction of function and KEGG Level 1 and Level 2 pathways of 7^th^ day. **B** *K. quasipneumoniae* abundance was associated with the most relevant pathways in mice of BLM-induced lung fibrosis at KEGG_Level 3 of 7^th^ day, as revealed by Tax4Fun analysis. **C**–**F** Differential analysis of KEGG pathway based on predicted metagenomes at different times. *n* = 3 per group.

The metagenome prediction tool Tax4Fun was used to extract functional annotations from the 16S rDNA gene sequences. On day 7, KEGG level 2 pathways involved in signal transduction, carbohydrate metabolism, and amino acid metabolism were dominant. By days 14, 21, and 28, signal transduction pathways became more prominent (Figure S3). At KEGG level 3, *K. quasipneumoniae* abundance was linked to key pathways involved in BLM-induced lung fibrosis, such as the AMPK, HIF-1, VEGF, PI3K-Akt, and NF-kappa B signaling pathways, as well as bacterial invasion of epithelial cells, ECM-receptor interaction, and IL-17 signaling (Fig. 4B and Supplementary Fig. S1). Meanwhile, there was a significant difference in signaling pathways at different times (Fig. 4C–F, *P* < 0.5).

**Fig. 5:**
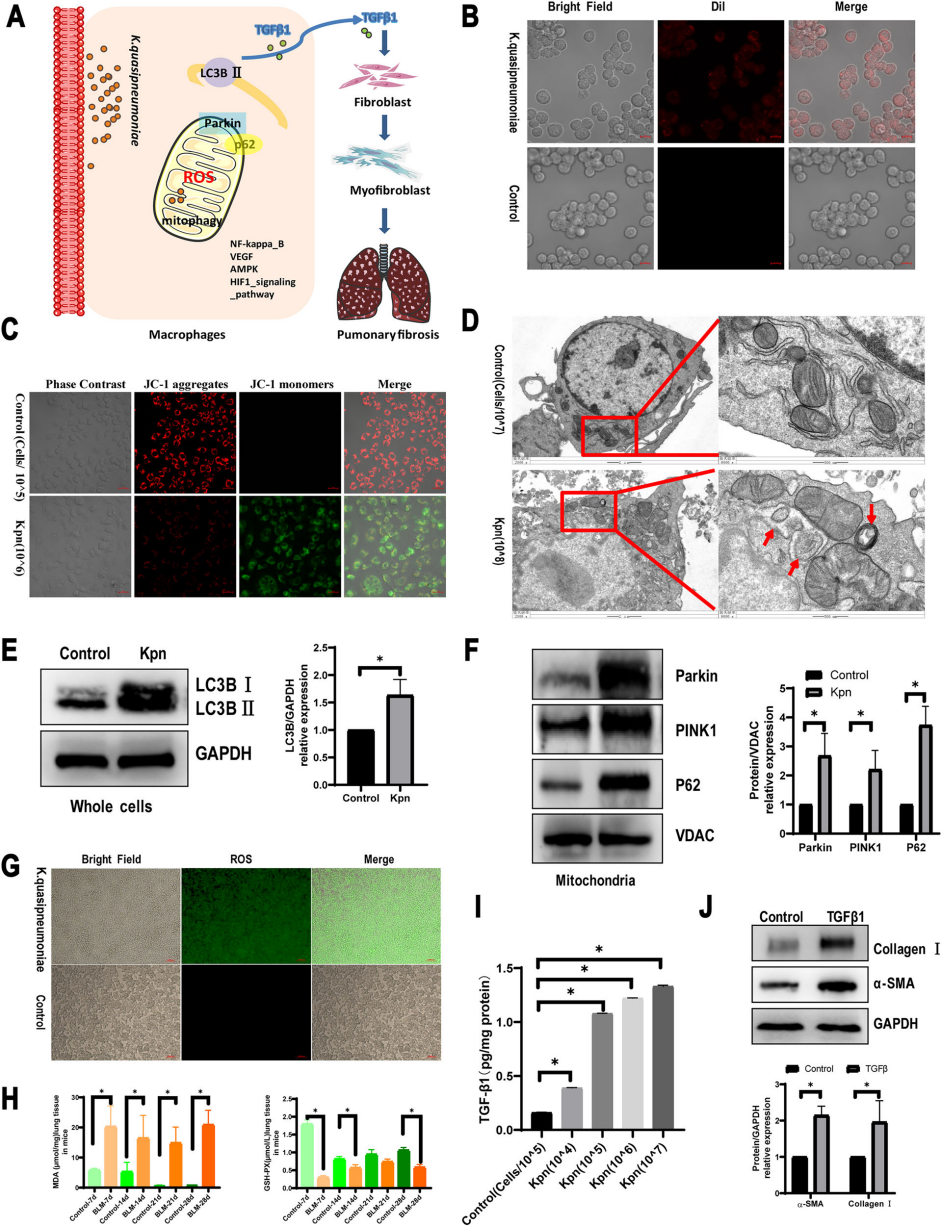
Macrophage mitophagy induced by *K. quasipneumoniae* modulates PF. **A** The diagram of mechanisms by which macrophage mitophagy induced by *K*. *quasipneumoniae* modulates PF. **B** Tracking *K. quasipneumoniae* in vitro. Dil red fluorescent dye (Dil: excitation = 549 nm; emission = 565 nm) was applied to label *K. quasipneumoniae* ATCC700603. **C** JC-1 staining images of vehicle, and the relative count of *K. quasipneumoniae* ATCC700603 to RAW264.7 cells is 10. Scale bars, 20 μm. **D** Ultrastructure of mitochondria in RAW264.7 cells treated for 24 h, and the relative count of *K. quasipneumoniae* ATCC700603 to RAW264.7 cells is 10. Arrows indicate autophagosomes. **E**, **F** LC3B-I and -II expression and PINK, Parkin, p62 were determined in RAW264.7 cells. **G** ROS production was induced by *K. quasipneumoniae* ATCC700603 in RAW264.7 cells. **H** MDA concentration and GSH-PX assay of lung tissues in mice. **I** Active TGF-β1 was measured by ELISA induced by different concentration of *K. quasipneumoniae* ATCC700603 in RAW264.7 cells. **J** Collagen I and α-SMA expression were determined in NIH-3T3 cells. *P < 0.05 compared with the control group, *n* = 3 per group. Kpn indicated *K. quasipneumoniae*.

To further investigate the relationship between *K. quasipneumoniae* and PF, RAW264.7 cells were used in additional experiments. *K. quasipneumoniae* ATCC700603 was shown to induce macrophage mitophagy, resulting in TGF-β1 production, which promotes fibrosis (Fig. 5A). ROS-related pathways, including AMPK, HIF-1, VEGF, PI3K-Akt, and NF-kappa B, were implicated in disease progression (Fig. 5A). To assess whether *K. quasipneumoniae* induces mitochondrial autophagy, RAW264.7 cells were co-cultured with *K. quasipneumoniae* at ratios of 0.1, 1, and 10. A ratio of 10 significantly decreased mitochondrial membrane potential (Fig. 5C and Supplementary Fig. S4B). Transmission electron microscopy revealed that *K. quasipneumoniae*-treated alveolar macrophages exhibited irregular mitochondrial morphology with disorganized cristae and empty areas (Fig. 5D and Supplementary Fig. S4A). Based on these results, a ratio of 10 was selected for subsequent experiments. Increased LC3B expression confirmed the induction of autophagy, and mitochondrial accumulation of PINK1, Parkin, and p62 was also observed (Fig. 5E, F). ROS generation was accompanied by changes in MDA and GSH-PX levels in lung tissue (Fig. 5G, H). ELISA measurements showed that *K. quasipneumoniae* treatment increased TGF-β1 expression in RAW264.7 cells. NIH-3T3 cells treated with 5 ng/mL TGF-β1 displayed increased expression of fibroblast differentiation markers, including collagen I and α-SMA, compared to controls. Collectively, these data suggest that *K. quasipneumoniae* promotes fibrosis progression in mice through macrophage mitophagy.

## Discussion

In this study, we conducted a comprehensive analysis of the respiratory tract microbiome in mice with BLM-induced PF. Our findings demonstrate that BLM exposure leads to lung microbiota dysbiosis and promotes fibrosis. Through 16S rDNA sequencing of the V3-V4 region, we observed a persistent shift in the proportion of *K. quasipneumoniae* in the lung microbiota, which increased as the disease progressed. This increase in *K. quasipneumoniae* was associated with heightened ROS production in macrophages, potentially contributing to the progression of PF. Collectively, our data provide strong evidence that lung microbiota dysbiosis plays a critical role in BLM-induced PF.

O’Dwyer et al. previously showed that the respiratory tract harbors dynamic microbial communities [4], with the microbiota of healthy individuals remaining relatively stable despite variations in pH, temperature, and oxygen levels [13]. The most common phyla in healthy airways include *Bacteroidetes*, *Firmicutes*, *Proteobacteria*, and *Actinobacteria* [13]. The composition of the lung microbiota is influenced by three primary factors: microbial immigration (via microaspiration, inhalation, and mucosal dispersion), microbial elimination (through mucociliary clearance, coughing, and immune defenses), and the local growth environment (involving nutrient availability, oxygen tension, pH, and temperature). These factors maintain a balance between microbial influx, efflux, and reproduction, which may fluctuate under pathological conditions. Studies have shown that the lung microbiome of patients with lung diseases, including IPF, differs significantly from that of healthy individuals [14]. In patients with IPF, alpha diversity decreases, and both species number and relative abundance (beta diversity) are altered [15]. In our study, we observed dynamic fluctuations in the lung microbiome at the phylum, genus, and species levels in BLM-induced PF mice, suggesting that changes in lung function may be driven by the expansion of certain species with competitive advantage and/or the loss of others [16].

Macrophages play a vital role in fibrosis initiation, maintenance, and resolution [17]. They are key contributors to IPF by producing profibrogenic factors, promoting cell growth, facilitating collagen formation, and supporting wound healing [18]. Anti-fibrotic macrophages secrete pro-inflammatory cytokines and eliminate pathogens, while pro-fibrotic macrophages exhibit anti-inflammatory properties, support angiogenesis and tissue remodeling, and are often associated with fibrotic conditions like PF [9]. Recent advances in immunology have demonstrated that innate macrophage activation by pathogens or pathogen-derived molecules is critical in the development of lung inflammation and fibrosis.

ROS are essential for bacterial clearance in the lung, and macrophages can enhance ROS production in response to changes in the microbiota [19]. Excess ROS can lead to mitochondrial depolarization, triggering mitophagy, which is crucial for maintaining mitochondrial and cellular homeostasis [20]. Mitophagy plays an important role in IPF, especially in activating lung fibroblasts via transforming growth factor-β (TGF-β) [20], which is consistent with our findings (Figs. 2D and 5I). Although lung remodeling during PF is poorly understood, the generation of ROS particularly mitochondrial H_2_O_2_ produced by alveolar macrophages, contribute to fibrosis development by increasing TGF-β expression [21]. ROS, although an important defense mechanism, can cause significant collateral damage or exacerbate pre-existing lung injuries, including PF [19]. *K. quasipneumoniae*, a normal component of the human microbiota, has been identified as a common pathogen in IPF patients with bacterial pneumonia [22, 23]. Changes in *K. quasipneumoniae* proportions in the lung microbiome may induce ROS production, leading to mitophagy and promoting fibrosis. In our study, the persistent presence of *K. quasipneumoniae*, as observed through fluorescence in situ hybridization, correlated with PF progression, suggesting that its expansion may contribute to fibrosis via mitophagy.

The advent of high-throughput techniques for identifying and studying respiratory bacteria has revolutionized our understanding of chronic lung diseases. Studies on the lung microbiome in PF have made significant contributions to this field, but much remains to be explored. Future research should focus on mechanistic studies examining the host response to microbiota dysbiosis and the interactions within bacterial communities in the lung. Understanding these complex interactions will pave the way for microbiome-based therapies for PF patients.

## Methods

### Animal model and ethics statement

Male C57BL/6J mice (6–8 weeks old) were obtained from Experimental Laboratory Animal Technology Co., Ltd. (SPF, Beijing, China). The mice were randomly assigned to eight groups using a double-blind method (*n* = 9 per time point in each group): Control (7, 14, 21, and 28 days) and BLM groups (7, 14, 21, and 28 days). Mice in the BLM groups received 5 mg/kg BLM (Nippon Kayaku, Tokyo, Japan), dissolved in 0.05 mL sterile saline, via intratracheal instillation. Control groups were treated with an equal volume (0.05 mL) of sterile saline. Mice were sacrificed at their designated time points (7, 14, 21, and 28 days), and alveolar lavage fluid and lung tissue were collected. Lung function was measured following previously published protocols. Histological analysis was conducted using hematoxylin-eosin (HE) staining, while collagen deposition was assessed via Masson’s staining and immunohistochemistry (IHC) for α-SMA (1:50, ab7817) and collagen I (1:100, sc293182). The Ashcroft score and fibrotic area were used to evaluate the severity of pulmonary fibrosis. Hydroxyproline (Hyp) content in lung tissue was measured using commercial detection kits (Nanjing Jian Cheng Biotechnology Research Institution, Nanjing, China).

### 16S rDNA sequencing analysis

The samples of alveolar lavage fluid were collected under sterile conditions to prevent exogenous contamination. Total genomic DNA was extracted using the CTAB/SDS method. Specific primers were used to amplify the 16S rDNA gene, and sequencing libraries were prepared with the Ion Plus Fragment Library Kit (Thermo Fisher, USA). Sequencing was performed on the IonS5™ XL platform (Thermo Fisher, USA). Negative controls were included in each PCR reaction. Quality filtering ensured high-quality reads. Alpha diversity indices (Shannon and Simpson) were used to assess species complexity within samples [24], while beta diversity was used to evaluate differences between samples [25].

### *Klebsiella quasipneumoniae* 16S RNA expression by RNA ISH assay

Paraffin-embedded lung sections (5 μm) were processed for RNA detection in situ using the RNAscope Multiplex Fluorescent Detection Reagent v2 (Advanced Cell Diagnostics, Hayward, CA). Double-Z oligonucleotide probes were designed to detect *K. quasipneumoniae* (16S rRNA, region 776–819). In brief, 4% formalin-fixed, paraffin-embedded lung sections were deparaffinized, dehydrated, and treated with peroxidase block for 10 min at room temperature. The sections were then pre-treated with a boiling solution for 15 min and proteinase K for 30 min at 40 °C. Hybridization with the *K. quasipneumoniae* 16S rRNA probe was conducted for 2 h at 40 °C in a HybEZ oven. Signal amplification and washing steps followed. Fluorescence labeling was performed using TSA Vivid 570 for 30 min at 40 °C, after which the sections were rinsed and counterstained with DAPI. Images were captured using laser-scanning confocal fluorescence microscopy (Zeiss, CA, Germany).

### Bacterial tracking and phagocytosis

*K. quasipneumoniae* ATCC700603 (American Type Culture Collection, Manassas, VA, USA) was labeled with fluorescent Dil (Beyotime Biotechnology, Shanghai, China). RAW264.7 macrophages (from Xiang Ya Central Experiment Laboratory, China) were cultured in DMEM (Hyclone, Beijing, China) supplemented with 10% fetal bovine serum (FBS, Gibco, Australia) and incubated at 37 °C in a 5% CO2 environment. At 80% confluence, the cells (1 × 10^5^) were incubated with *K. quasipneumoniae* ATCC700603 (1 × 10^6^ CFU/mL), opsonized in normal serum at 37 °C for 1 h. Gentamicin (10 mg/mL) was then added to eliminate extracellular bacteria. The cells were washed in antibiotic-free PBS, and the macrophages were lysed immediately or after 2 h on ice using distilled water.

### Transmission electron microscopy

Macrophages were fixed in 2.5% glutaraldehyde in Sorenson’s phosphate buffer (pH 7.0). After processing and sectioning at 70–90 nm with a diamond knife, sections were mounted on copper mesh grids, stained with uranyl acetate and lead citrate, and examined using a JEM-1200EX transmission electron microscope (Hitachi, Tokyo, Japan).

### Reactive oxygen species (ROS) measurement

ROS levels were determined using the 2′, 7′-dichlorofluorescin diacetate (DCFH-DA) assay (Beyotime Institute of Biotechnology, Shanghai, China). RAW264.7 cells (≥1 × 10^6^) were incubated with 1 mM DCFH-DA at 37 °C for 30 min. After washing, cells were resuspended in PBS, and ROS accumulation was measured by fluorescence microscopy (Zeiss, CA, Germany) at 485/530 nm.

### Lipid peroxidation (MDA) and glutathione peroxidase (GSH-PX) assays

The generation of ROS may be accompanied by changes in malondialdehyde (MDA) and glutathione peroxidase (GSH-PX). MDA levels in lung tissue were measured using a Lipid Peroxidation MDA Assay Kit (Beyotime Institute of Biotechnology, Shanghai, China). GSH-PX activity was assessed using a colorimetric assay kit (Nanjing Jian Cheng Biotechnology Research Institution, Nanjing, China).

### Statistical analysis

Data were analyzed using IBM SPSS software version 21.0 (IBM Corp., Armonk, NY, USA). Graphs were generated with GraphPad Prism 8.0 (GraphPad Software, San Diego, CA, USA). Results were expressed as mean ± SD. For comparisons between two groups, Student’s *t* test was used, while one-way ANOVA was applied for comparisons among multiple groups. All experiments were conducted independently three times, and all tests were two-tailed. A *p*-value of less than 0.05 (*P* < 0.05) was considered statistically significant.

## Supplementary information

Primitive of WB

supplementary

## Data Availability

All data relevant to the study are included in the article or uploaded as supplementary information.
