# Supplementary figures and images for: Tissue-resident Klebsiella quasipneumoniae contributes to progression of idiopathic pulmonary fibrosis by triggering macrophages mitophagy in mice
Source: Cell Death Discov. 2025 Apr 12;11:168. doi: 10.1038/s41420-025-02444-6 (PMC11993561; doi:10.1038/s41420-025-02444-6)

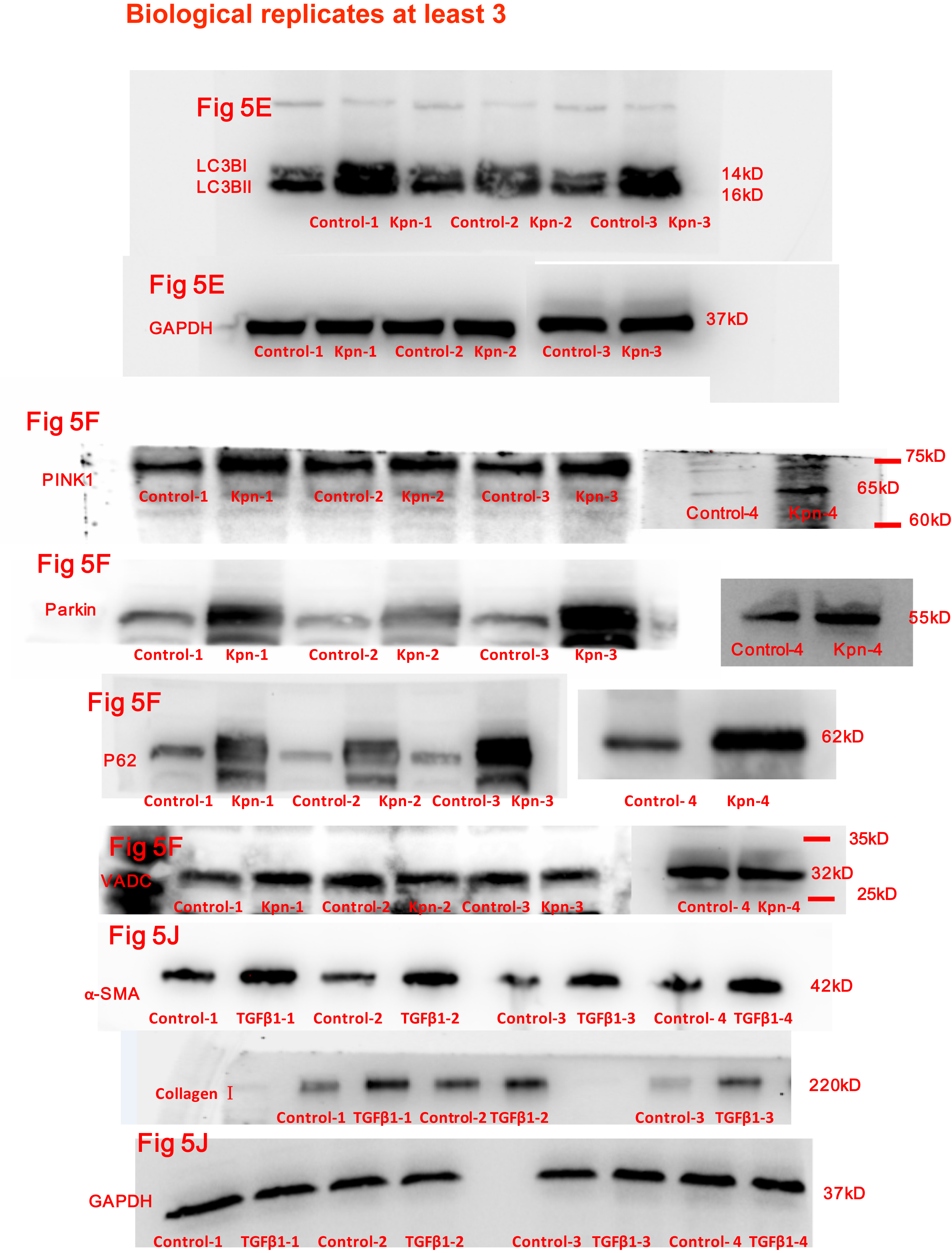

Supplement: Supplementary file 1 — Primitive of WB [file 41420_2025_2444_MOESM1_ESM.tif]
