# Supplementary material for: Tissue-resident Klebsiella quasipneumoniae contributes to progression of idiopathic pulmonary fibrosis by triggering macrophages mitophagy in mice
Source: Cell Death Discov. 2025 Apr 12;11:168. doi: 10.1038/s41420-025-02444-6 (PMC11993561; doi:10.1038/s41420-025-02444-6)
Supplement: Supplementary file 2 — supplementary [file 41420_2025_2444_MOESM2_ESM.docx]

**
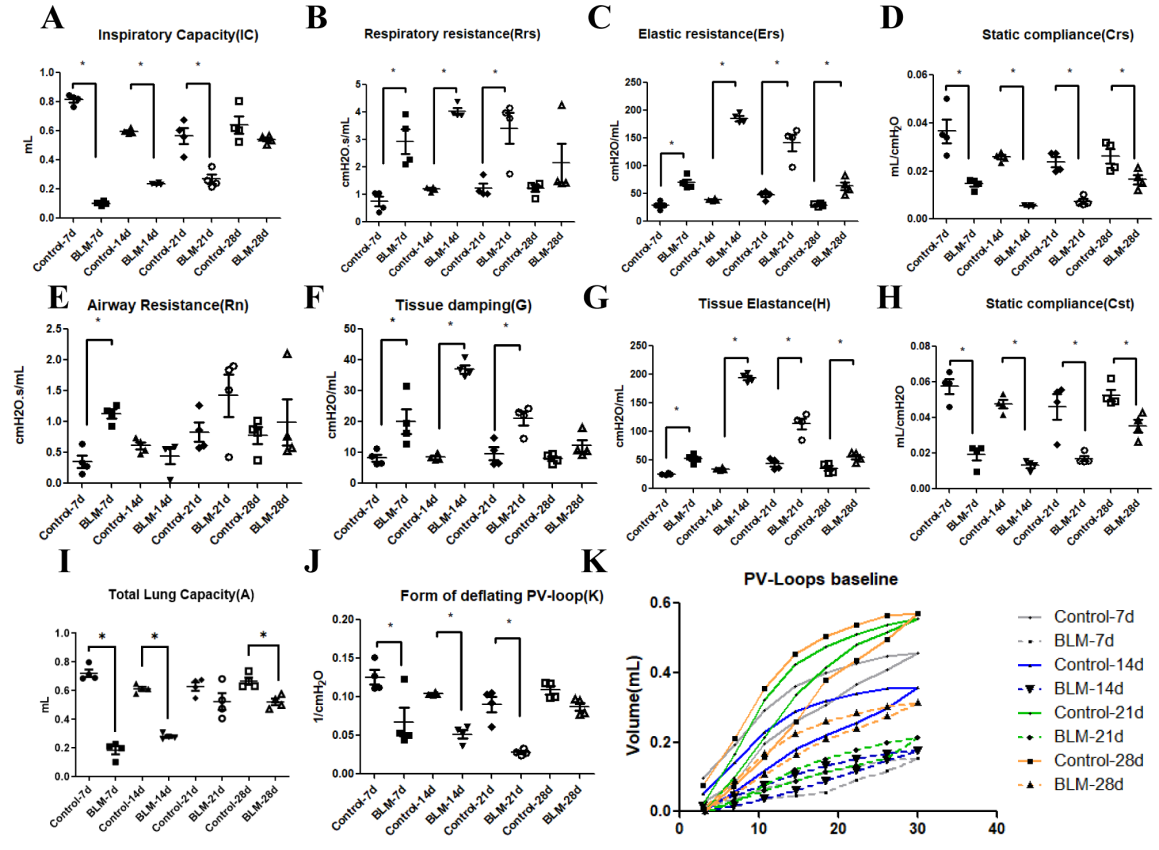
**

Figure S1 Respiratory function of BLM-induced mice at different times

(A) Inspiratory capacity was performed by the model of Deep Inflation. (B-D) Respiratory resistance, elastic resistance, and static compliance were measured by the model of Snap Shot. (E-G) Airway resistance, tissue damping, and tissue elastance were performed by the Quick Prime. (H-K) PV loops were applied to assess static compliance, total lung capacity, and form of deflating PV loop. PV loop baseline was performed by the PV loops. This was separately calculated for each group. The data are presented as means ± SD (n = 4). **P* < 0.05 when compared with the control group.

We assessed the effect of BLM-induced PF on lung function at different times using forced oscillation technique (FOT), which has been applied to mouse models of respiratory diseases to investigate pathophysiological changes associated with fibrosis[^12^](#_ENREF_12). Once the manoeuvre was initiated, the *flexiVent* (SCIREQ, Montreal, Canada) was programmed to gradually inflate the mouse lungs to a pressure of 30 cm H_2_O over 1 second, before opening the shutter valve, and hold this pressure for 2 seconds to connect the animal's airway opening to the negative pressure reservoir for 2 seconds. The negative pressure gradient generated a rapid deflation of the mouse lungs, and the ensuing flow of air into the body box associated with the animal chest wall movement was measured. The *flexiVent* system included four models: the basic model of total lung capacity (TLC) (Figure S1A), Snap Shot (Figure S1B-D), Quick Prime (Figure S1E-G), and the pressure-volume (PV) loop (Figure S1H-K) model.

Inspiratory capacity (IC) of mice exhibited decline on days 7, 14 and 21 after exposure to BLM (Figure S1A). The Snap Shot model was used to access mechanics of the respiratory system, and the inspiratory capacity, normalized by body weight, appeared to be similar in the three groups (Figure S1A). Compared with the control group, BLM increased the respiratory resistance (Rrs) (except 28^th^ day) and elastic resistance (Ers), and suppressed static compliance (Crs) at different times (Figure S1B-D). The Quick Prime module was applied to distinguish airway resistance and lung resistance. BLM-induced instillation increased the central airway resistance (Rn) on day 7 only, yet tissue damping (G) and tissue elastance (H) at different times (Figure S1E-G). The PV loop module was estimated including the measurements of total lung capacity (A), static compliance (Cst), form of deflating PV loop (K), and PV loop baseline (Figure S1H-K). Notably, the PV loops of mice in the BLM group revealed a significant downward at different times (Figure S1H-K). However, it could be seen that these effects declined on day 28, compared to other times. The possible reason is that the mice with severe fibrosis developed breathing difficulties over time and died on that day.


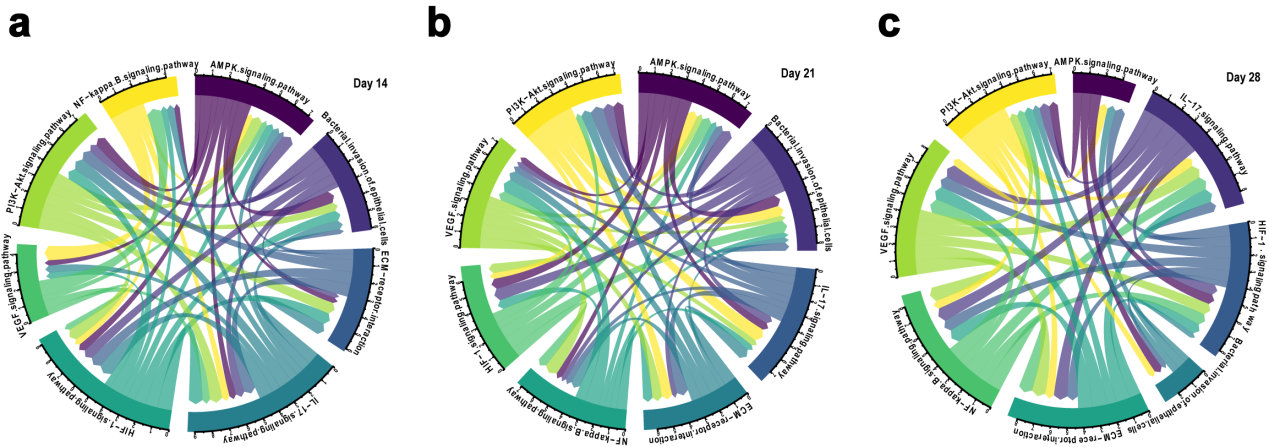
Figure S2 The prediction of function and KEGG level1 and level2 pathways at 14, 21 and 28 days.


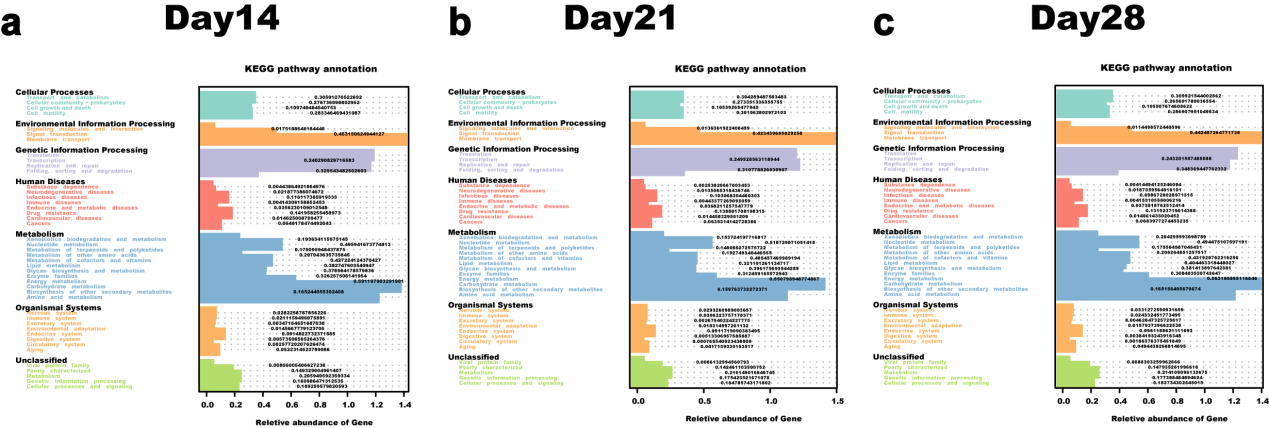
Figure S3. *K. quasipneumoniae* abundance was associated with the most relevant pathways in mice of BLM-induced lung fibrosis at KEGG_Level3 of 14^th^ day, 21^th^ day and 28^th^ day, as revealed by Tax4Fun analysis.


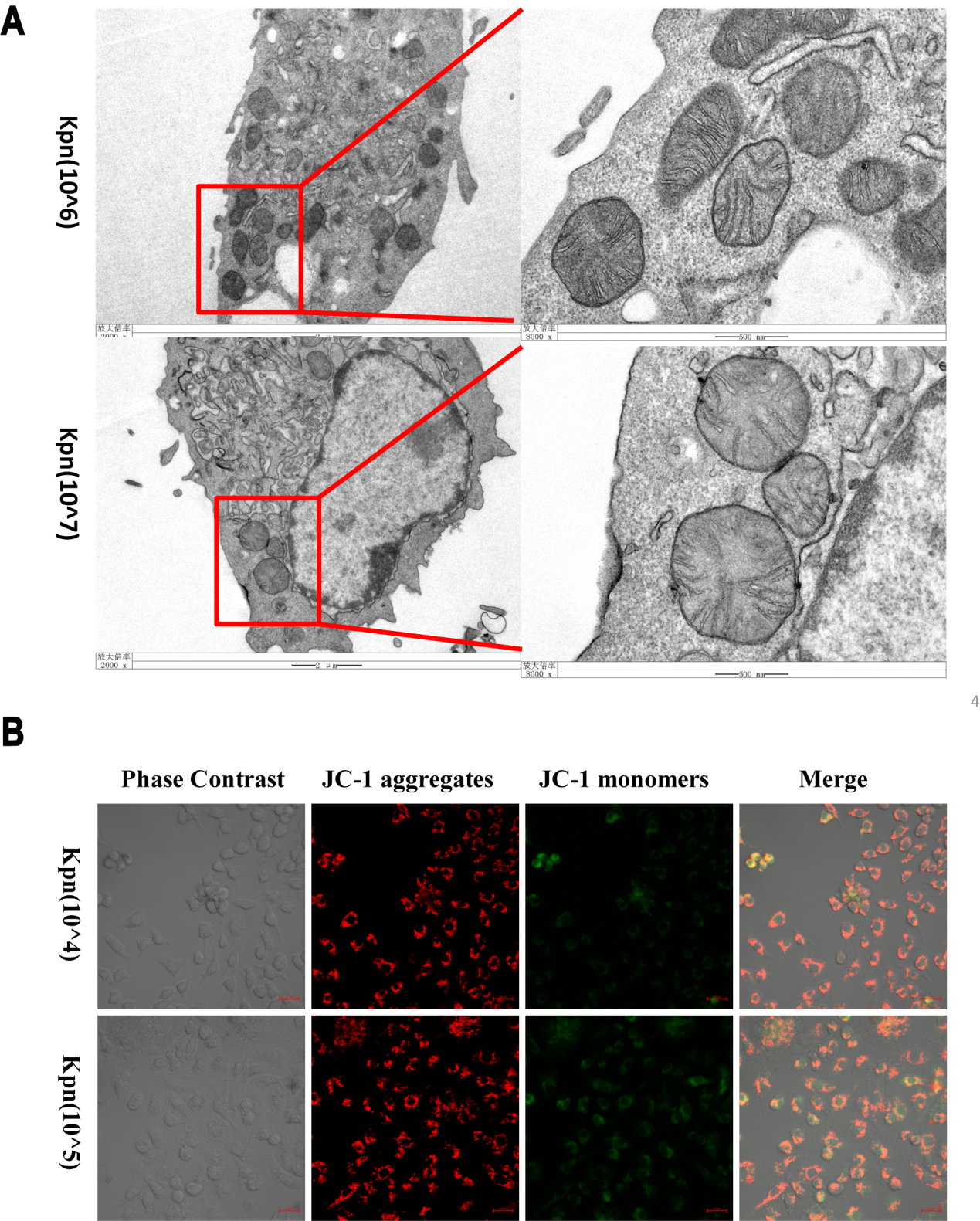


Figure S4 JC-1 staining images of vehicle and Ultrastructure of mitochondria. The relative count of *K. quasipneumoniae* to RAW264.7 cells is -1and 1.

**Western blotting**

Total proteins of tissues and cells were solubilized in a mixture of RIPA, protease inhibitor, and phosphatase inhibitor (Beyotime Institute of Biotechnology, China). BCA protein analysis kit (Invtrogen, USA) was used to detect protein concentrations. 30 mg of protein samples were separated by 10% SDS-PAGE and then transferred to PVDF membranes (EMD Millipore). Use 5% nonfat milk powder to cover for 1 hour, then put the membranes into the antibody against Parkin (1:1000; ab77924), PINK1 (1:1000; 23274-1-AP), VDAC (1:1000; 4661T), P62 (1:1000; ab109012), GAPDH (1:3000; 2118T), at 4 °C overnight.
